# Cluster-Based White Matter Signatures and the Risk of Dementia, Stroke, and Mortality in Community-Dwelling Adults

**DOI:** 10.1212/WNL.0000000000209864

**Published:** 2024-09-10

**Authors:** Mathijs T. Rosbergen, Frank J. Wolters, Elisabeth J. Vinke, Francesco U.S. Mattace-Raso, Gennady V. Roshchupkin, Mohammad Arfan Ikram, Meike W. Vernooij

**Affiliations:** From the Department of Epidemiology (M.T.R., F.J.W., E.J.V., F.U.S.M.-R., G.V.R., M.A.I., M.W.V.), Department of Radiology and Nuclear Medicine (M.T.R., F.J.W., E.J.V., G.V.R., M.W.V.), Department of Internal Medicine (F.U.S.M.-R.), and Department of Medical Informatics (G.V.R.), Erasmus MC University Medical Center, Rotterdam, the Netherlands.

## Abstract

**Background and Objectives:**

Markers of white matter (WM) injury on brain MRI are important indicators of brain health. Different patterns of WM atrophy, WM hyperintensities (WMHs), and microstructural integrity could reflect distinct pathologies and disease risks, but large-scale imaging studies investigating WM signatures are lacking. This study aims to identify distinct WM signatures using brain MRI in community-dwelling adults, determine underlying risk factor profiles, and assess risks of dementia, stroke, and mortality associated with each signature.

**Methods:**

Between 2005 and 2016, we measured WMH volume, WM volume, fractional anisotropy (FA), and mean diffusivity (MD) using automated pipelines on structural and diffusion MRI in community-dwelling adults aged older than 45 years of the Rotterdam study. Continuous surveillance was conducted for dementia, stroke, and mortality. We applied hierarchical clustering to identify separate WM injury clusters and Cox proportional hazard models to determine their risk of dementia, stroke, and mortality.

**Results:**

We included 5,279 participants (mean age 65.0 years, 56.0% women) and identified 4 distinct data-driven WM signatures: (1) above-average microstructural integrity and little WM atrophy and WMH; (2) above-average microstructural integrity and little WMH, but substantial WM atrophy; (3) poor microstructural integrity and substantial WMH, but little WM atrophy; and (4) poor microstructural integrity with substantial WMH and WM atrophy. Prevalence of cardiovascular risk factors, lacunes, and cerebral microbleeds was higher in clusters 3 and 4 than in clusters 1 and 2. During a median 10.7 years of follow-up, 291 participants developed dementia, 220 had a stroke, and 910 died. Compared with cluster 1, dementia risk was increased for all clusters, notably cluster 3 (hazard ratio [HR] 3.06, 95% CI 2.12–4.42), followed by cluster 4 (HR 2.31, 95% CI 1.58–3.37) and cluster 2 (HR 1.67, 95% CI 1.17–2.38). Compared with cluster 1, risk of stroke was higher only for clusters 3 (HR 1.55, 95% CI 1.02–2.37) and 4 (HR 1.94, 95% CI 1.30–2.89), whereas mortality risk was increased in all clusters (cluster 2: HR 1.27, 95% CI 1.06–1.53, cluster 3: HR 1.65, 95% CI 1.35–2.03, cluster 4: HR 1.76, 95% CI 1.44–2.15), compared with cluster 1. Models including clusters instead of an individual imaging marker showed a superior goodness of fit for dementia and mortality, but not for stroke.

**Discussion:**

Clustering can derive WM signatures that are differentially associated with dementia, stroke, and mortality risk. Future research should incorporate spatial information of imaging markers.

## Introduction

Cerebral white matter (WM) injury is a heterogeneous process of pathophysiologic changes, reflected by WM atrophy, WM hyperintensities (WMH), and loss of microstructural integrity on brain MRI. To assess these changes noninvasively, MRI is a useful technique using imaging sequences, such as T1-weighted imaging and T2-weighted fluid-attenuated inversion recovery (FLAIR) imaging for macrostructural properties. More recently, diffusion tensor imaging (DTI) has been proven to be a useful MRI technique in obtaining microstructural properties of cerebral WM. Fractional anisotropy (FA) and mean diffusivity (MD) are commonly used DTI measures which reflect the directional constraint and the magnitude of water diffusion.^1^ Previous research has established that these markers are all associated with the risk of dementia, stroke, and mortality.^2-8^ However, published studies mostly investigated individual imaging markers of WM, leaving undetermined how these imaging markers co-occur and how their interplay affects clinical outcome within individuals.

Novel data-driven techniques may aid to unravel WM patterns on the basis of multimodal imaging data, hereafter referred to as WM signatures. Studies focusing on cognitive decline and brain atrophy have used machine learning approaches on multimodal imaging markers.^9-11^ These studies showed that imaging-based clusters are associated with cognitive patterns both in a cross-sectional manner and in cognitive decline. Although these studies focused on imaging-based clusters, they included supplementary CSF markers in their clustering methodology or focused on a specific population and did not consider to primarily focus on noninvasively obtainable WM imaging markers within the general population.

Despite increasing literature regarding individual WM imaging markers and imaging-based clusters, it lacks comprehensive exploration into the diverse patterns of WM injury and how these patterns may reflect different underlying pathologies. Moreover, the extent to which these patterns of WM injury contribute to differential risks for dementia, stroke, and mortality remains largely unexplored.

The aim of this study was to identify distinct WM signatures using both structural and diffusion MRI in community-dwelling adults and determine underlying risk factor profiles. Moreover, we will assess the risks of dementia, stroke, and mortality associated with each of these signatures. Through this approach, we aim to unravel the origins and diverse manifestations of WM injury, ultimately contributing to a better understanding of their implications for health outcomes.

## Methods

### Study Population

This research was embedded in the Rotterdam Study, an ongoing prospective population-based cohort located in Rotterdam, the Netherlands.^12^ Since 1990, residents of the Ommoord district aged 45 years or older were invited to participate, with follow-up every 4 years. Participants underwent routine brain MRI from 2005 onwards. Changes to the diffusion imaging sequence were implemented in the first year; therefore, this study includes all participants with brain MRI between March 29, 2006, and June 29, 2016.

### Standard Protocol Approvals, Registrations, and Patient Consents

The Rotterdam Study received approval from the institutional review board (Medical Ethics Committee) of the Erasmus Medical Center and by the Ministry of Health, Welfare and Sports of the Netherlands, implementing the “Wet Bevolkingsonderzoek: ERGO (Population Screening Act: Rotterdam Study).” All participants provided written informed consent for their participation in the study.

### MRI Acquisition, Preprocessing, and Appraisal

Brain MRI scanning was conducted for all participants using a 1.5 Tesla MRI scanner (Signa Excite II; General Electric Healthcare, Chicago, IL) with an 8-channel head coil. All scans were acquired using a single scanner in which no changes in hardware, software, and scan protocol took place. The scan protocol and sequence details of the Rotterdam Study have been described elsewhere.^12^ For volumetry, T1-weighted (voxel size 0.49 × 0.49 × 1.6 mm^3^), proton density-weighted (voxel size 0.6 × 0.98 × 1.6 mm^3^), and FLAIR (voxel size 0.78 × 1.12 × 2.5 mm^3^) scans were used for automated segmentation of supratentorial WM, WMH, and intracranial volume.^13,14^ Two raters visually inspected and manually corrected the segmentations if necessary. When a rater was in doubt, both inspected the mask and reached a consensus. WM volume was defined as total supratentorial WM, including WMH. WM volume and WMH volume were divided by supratentorial intracranial volume to adjust for head size. For microstructural measures, diffusion tensor imaging (voxel size 3.3 × 2.2 × 3.5 mm^3^) was used to quantify global FA and global MD of normal-appearing WM. Data were processed using a standardized pipeline^15^ and combined with tissue segmentation, providing global FA and MD values in normal-appearing WM. Only voxels with FA ≥ 0.2 were included to minimize involvement of partial volume effects arising from gray matter or CSF.^15^

All scans were rated by trained researchers, blinded to clinical data, for the presence of lacunes, cortical infarcts, and microbleeds. Lacunes were defined as focal parenchymal lesions ranging from ≥3 mm to <15 mm in size with similar signal intensity as CSF on all sequences, and—when located supratentorial—with a hyperintense rim on FLAIR images.^16^ To differentiate lacunes from dilated perivascular spaces, symmetry of the lesions, sharp demarcation, and absence of a hyperintense rim on the FLAIR sequence supported the presence of a dilated perivascular space.^17^ Microbleeds were identified as focal areas with a very low intensity on a 3D T2*-weighted gradient-recalled echo MRI scan (voxel size 0.78 × 1.12 × 1.6 mm^3^), unaccompanied by signal abnormalities on other sequences.^18^

### Clinical Characteristics and Other Variables

Information regarding educational attainment, smoking habits, alcohol intake, and medication use was obtained by trained interviewers during baseline home interviews. Educational attainment was categorized into 4 groups, ranging from primary to higher education (higher vocational education or university). Smoking was categorized into 3 groups: former smoker, current smoker, or never smoker. Body mass index (BMI) was calculated as weight in kilograms divided by squared height in meters. Blood pressure was measured twice at the right arm with the participant in the seated position. The mean of 2 consecutive measurements was used. Serum total cholesterol and high-density lipoprotein (HDL) cholesterol were measured during visits at the research center. Diabetes mellitus was defined as a fasting blood glucose concentration of ≥7.0 mmol/L or as use of antidiabetic drugs. *APOE* genotype was determined using PCR or biallelic Taqman assay.

### Assessment of Dementia, Stroke, and Mortality

All participants were followed for the onset of dementia, stroke, and mortality until January 1, 2020, through a combination of repeated in-person assessment and linkage with electronic medical records. Follow-up for each participant started at the time of their MRI scan, which was between March 29, 2006, and June 29, 2016.

During each visit to the research center, participants underwent dementia screening with the Mini-Mental State Examination (MMSE) and the Geriatric Mental State Schedule (GMS) organic level. Participants with a MMSE <26 or a GMS >0 underwent an examination and informant interview with the Cambridge Examination for Mental Disorders of the Elderly. Continuous surveillance for dementia across the entire cohort was facilitated through electronic linkage of the study database with medical records from general practitioners and the Regional Institute for Outpatient Mental Health Care.^19^ A consensus panel, led by a consultant neurologist, decided on the final dementia diagnosis in all cases, in accordance with the *Diagnostic and Statistical Manual of Mental Disorders III-R* criteria. Participants were censored at date of dementia diagnosis, death, loss to follow-up or January 1, 2020, whichever occurred first. Dementia follow-up was near complete until January 1, 2020 (91.2% of potential person years).

Definition of stroke adhered to the World Health Organization criteria,^20^ describing a syndrome characterized by the rapid onset of focal or global cerebral dysfunction lasting 24 hours or longer or leading to death, with apparent vascular cause. Prevalent stroke was assessed at baseline during interview, and we verified these data with medical records from the general practitioner. In the Netherlands, general practitioners serve as gatekeepers and have access to comprehensive clinical data for their patients. From baseline onward, we maintained continuous surveillance for incident stroke through linkage of the study database with files from general practitioners. In addition, we checked files of nursing home physicians and files from general practitioners of participants who moved out of the district were also checked. We obtained additional information from hospital records. When potential strokes were found, they were reviewed by research physicians and verified by an experienced neurologist. Subarachnoid hemorrhages attributed to ruptured aneurysms were excluded. Participants were censored at date of stroke diagnosis, death, loss to follow-up, or January 1, 2020, whichever occurred first. Stroke follow-up was near complete until January 1, 2020 (97.8% of potential person years).

We assessed all-cause mortality by obtaining information on vital status from municipal records and clinical follow-up data collection. Coded information on all-cause mortality until January 1, 2020, was used for this study.

### Data Analysis

Missing data on covariables (maximum 7.0%) were imputed using 5-fold multiple imputation. Distribution of variables was similar in the imputed and nonimputed data sets.

We performed quantile regression to obtain age-adjusted measures of global FA, global MD, WMH volume, and WM volume by predicting 1,000 quantiles of the regression function for each imaging marker (ranging from 0 to 1), and assigning each individual to their closest predicted quantile. This results in higher quantile scores for individuals with high values of an imaging marker, compared with their counterparts of the same age. We accounted for nonlinearity in the trajectories by using natural cubic splines of age with 1 knot for all imaging markers.

To identify different WM signatures, we then performed unsupervised hierarchical agglomerative clustering with age-adjusted global FA, global MD, WMH volume, and WM volume as input parameters for each participant. As sensitivity analysis, we repeated this clustering method without adjusting these baseline imaging markers for age before clustering. Within agglomerative hierarchical clustering, each individual starts as a cluster of 1 person, after which clusters are iteratively merged based on similarity, determined by maximizing or minimizing an objective function and a metric for distance between individuals based on their imaging marker quantiles. This approach will result in every participant being clustered into only one of the clusters, and no participant will be left unclassifiable. We assessed cluster tendency in our data using the Hopkins statistic, which provides a value ranging from 0 (uniformly distributed data) to 1 (highly clustered data). The Hopkins statistic was 0.86, indicating a clustered distribution of WM pathology in the cohort, persisting after assigning age quantiles. We then performed hierarchical clustering, which is an effective method for data sets that are not excessively large and where the inherent structure is not overly complex. We used Ward linkage as an objective function aiming to minimize the total within-cluster variance and to create internally cohesive and well-defined clusters. Euclidean distance was adopted as the distance metric because it is the most common and intuitively straightforward distance measure. To determine the optimal number of clusters, we used the R package NbClust, which applies various cluster validity metrics, such as the gap statistic, Dunn index, and Silhouette score, to assess validity for different cluster numbers. The optimal number is determined by selecting the number with the highest frequency of metrics suggesting its optimum cluster validity. After performing the clustering, we assessed the stability of clusters by using bootstrapping, generating 100 subsamples (90% of the original data set) for repeating the clustering analysis on each subsample. The Adjusted Rand Index, ranging from −1 to 1, was then calculated to evaluate the consistency with which individuals were clustered together across the subsamples, with higher values indicating a greater degree of agreement between clustering results compared with random chance. The Adjusted Rand Index was calculated to compare the cluster labels of every subsample with the cluster labels of the full sample. Subsequently, we calculated the mean Adjusted Rand Index across all subsamples.

Next, we compared several participant characteristics between clusters, using *t* tests for continuous variables and χ^2^ tests for categorical variables. We used Cox proportional hazard models to obtain hazard ratios (HRs) and 95% CI for the association between clusters and risk of dementia, stroke, and mortality. For all Cox models, we tested the proportional hazard assumption using Schoenfeld residuals. We constructed 3 different models: a crude analysis (model 1); an analysis adjusted for age, sex, and education (model 2); and an analysis with additional adjustment for *APOE* genotype, lacunes, microbleeds, and cardiometabolic risk factors, including BMI, smoking status, alcohol intake, systolic blood pressure, diastolic blood pressure, use of blood pressure-lowering drugs, diabetes, serum total cholesterol, serum HDL, and use of lipid-lowering drugs. In sensitivity analyses, we (1) additionally adjusted for gray matter volume and (2) compared the associations of the clusters with clinical outcomes with a similar model assessing each imaging marker individually. Performance of the models was compared using the Akaike Information Criterion (AIC). All analyses were performed using R, version 4.1.2 (packages: fmsb, gplots, gridExtra, hopkins, mice, mclust, NbClust, quantreg, sjmiscc, survival). This study adhered to the Strengthening the Reporting of Observational Studies in Epidemiology guidelines for observational studies.^21^

### Data Availability

Requests for the anonymized data underlying this report can be directed to data manager Frank J.A. van Rooij (secretariat.epi@erasmusmc.nl).

## Results

**Figure 1:**
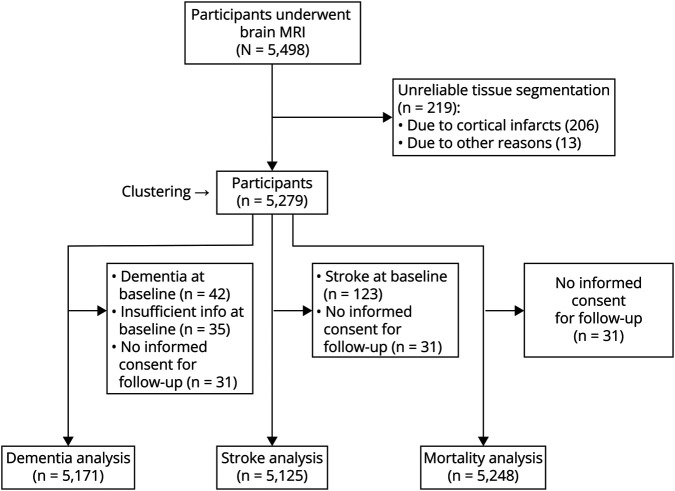
Flowchart of the Inclusion of Participants

During the study period, 5,498 participants underwent brain MRI, of whom 5,279 could be included in this study (Figure 1). Baseline characteristics of all participants included in the clustering analyses are presented in Table 1. The mean age at time of MRI scan was 65.0 years, and 56.0% of the participants were women.

**Table 1:** Characteristics of the Total Study Population and White Matter Clusters

Characteristic	Total (N = 5,279)	Cluster 1 (N = 1,457)	Cluster 2 (N = 1,791)	Cluster 3 (N = 956)	Cluster 4 (N = 1,075)	*p* Value
Age, y	65.0 (11.0)	65.0 (11.0)	64.9 (11.0)	65.6 (11.0)	64.9 (10.8)	0.424
Sex, female	2,957 (56.0)	777 (53.3)	945 (52.8)	618 (64.6)	617 (57.4)	<0.001
Education						0.038
Primary only	470 (8.9)	109 (7.5)	176 (9.8)	96 (10.0)	89 (8.3)	
Lower vocational	2,018 (38.2)	556 (38.2)	659 (36.8)	388 (40.6)	415 (38.6)	
Intermediate vocational/higher general	1,594 (30.2)	470 (32.3)	550 (30.7)	250 (26.2)	324 (30.1)	
Higher vocational/university	1,197 (22.7)	322 (22.1)	406 (22.7)	222 (23.2)	247 (23.0)	
Body mass index, kg/mm^2^	27.4 (4.1)	27.1 (3.7)	27.7 (4.2)	27.3 (4.2)	27.7 (4.3)	<0.001
Systolic blood pressure, mm Hg	139.6 (21.4)	137.7 (21.5)	139.2 (20.6)	141.3 (22.3)	141.2 (21.4)	<0.001
Diastolic blood pressure, mm Hg	82.5 (11.0)	81.5 (10.7)	82.2 (10.7)	83.6 (11.4)	83.5 (11.5)	<0.001
Blood pressure lowering medication	1,869 (35.4)	432 (29.6)	603 (33.7)	387 (40.5)	447 (41.6)	<0.001
Smoking						0.056
Never	1,622 (30.7)	439 (30.1)	545 (30.4)	314 (32.8)	324 (30.1)	
Former	2,601 (49.3)	736 (50.5)	913 (51.0)	440 (46.0)	512 (47.6)	
Current	1,056 (20.0)	282 (19.4)	333 (18.6)	202 (21.2)	239 (22.2)	
Alcohol intake,^a^ g/d	6.43 (0.54–10.00)	6.43 (0.57–8.57)	6.43 (1.25–14.93)	6.43 (0.54–8.57)	6.43 (0.71–11.93)	<0.001
Total cholesterol, mmol/L	5.53 (1.05)	5.57 (1.01)	5.54 (1.07)	5.53 (1.07)	5.47 (1.05)	0.111
HDL cholesterol, mmol/L	1.45 (0.42)	1.46 (0.41)	1.43 (0.42)	1.48 (0.43)	1.45 (0.43)	0.006
Lipid lowering medication	1,291 (24.6)	353 (24.2)	420 (23.5)	233 (24.4)	299 (27.8)	0.089
Diabetes mellitus	675 (12.8)	147 (10.1)	235 (13.1)	126 (13.2)	167 (15.5)	0.001
*APOE* genotype						0.097
ε3/ε3	3,002 (56.9)	844 (57.9)	1,034 (57.7)	537 (56.2)	587 (54.6)	
ε2/ε2 or ε2/ε3	876 (16.6)	227 (15.6)	288 (16.1)	163 (17.1)	198 (18.4)	
ε4-carrier (heterozygous)	1,294 (24.5)	363 (24.9)	438 (24.5)	225 (23.5)	268 (24.9)	
ε4-carrier (homozygous)	107 (2.0)	23 (1.6)	31 (1.7)	31 (3.2)	22 (2.0)	
Cerebral microbleeds (any)	1,063 (20.1)	257 (17.6)	311 (17.4)	247 (25.8)	248 (23.1)	<0.001
Lacunes (any)	396 (7.5)	57 (3.9)	109 (6.1)	104 (10.9)	126 (11.7)	<0.001
Gray matter volume, proportion of ICV	0.47 (0.03)	0.46 (0.03)	0.48 (0.03)	0.45 (0.03)	0.47 (0.03)	<0.001

Abbreviations: HDL = high-density lipoprotein; ICV = intracranial volume.

Continuous variables are presented as mean (SD) and categorical variables as n (%).

*p* Values showing differences among cluster for each variable.

a Variable with skewed distribution. Median (interquartile range) is shown instead of mean (SD).

### WM Signatures

**Figure 2:**
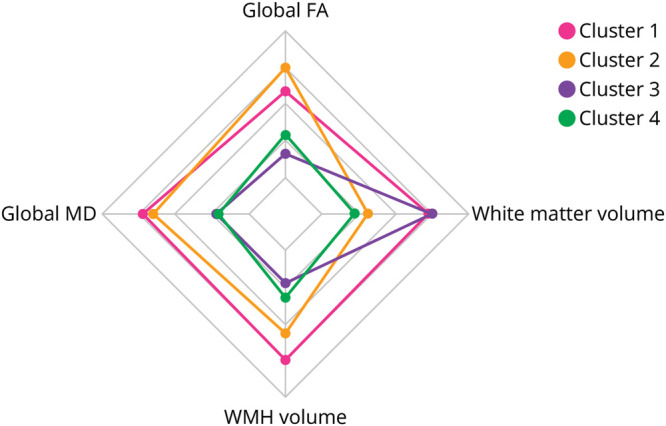
Composition of the Different White Matter Clusters Radar plot showing median values of white matter imaging markers for each cluster. Outer rim values represent favorable values (i.e., smaller WMH volume, larger white matter volume, higher global FA, and lower global MD). FA = fractional anisotropy; MD = mean diffusivity; WMH = white matter hyperintensities.

**Figure 3:**
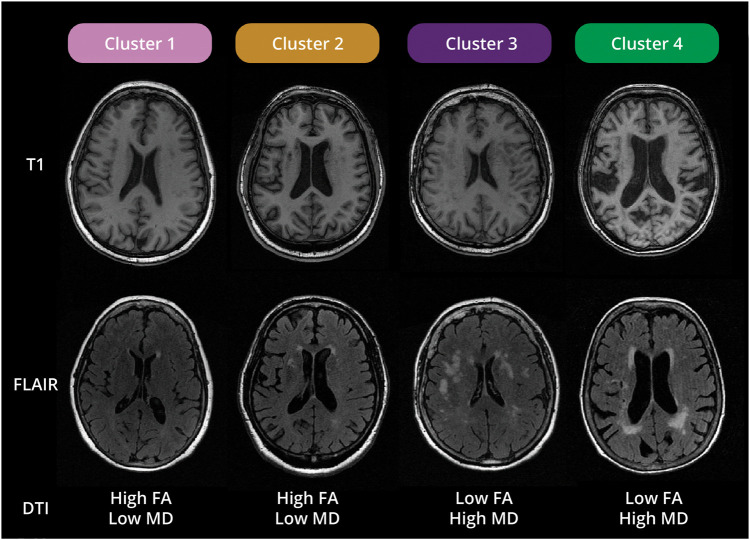
Typical MR Images of the Brain for Each Cluster For each cluster, MR images of a single participant in that cluster are shown to demonstrate typical MR images of the brain for each cluster. Microstructural properties derived from DTI were shown as text rather than images since diffusion-weighted images for fractional anisotropy and median diffusivity are difficult to interpret intuitively. DTI = diffusion tensor imaging; FA = fractional anisotropy; FLAIR = fluid-attenuated inversion recovery; MD = mean diffusivity.

We identified 4 different clusters of WM characteristics (eFigures 1 and 2), including between 956 and 1,791 individuals each. The acquired dendrogram and results of determining the optimal number of clusters are presented in the Supplementary Materials. Four distinct WM signatures emerged (Figure 2). Participants in cluster 1 had above-average microstructural integrity and little WM atrophy and WMH. Participants in cluster 2 had equally above-average microstructural integrity and an average WMH burden, but with substantial WM atrophy. Participants in cluster 3 had poor microstructural integrity and a relatively high burden of WMH but displayed little WM atrophy. Participants in cluster 4 also had poor microstructural integrity, yet with substantial WM atrophy and WMH burden. These characteristics can also be seen in Figure 3, which shows MR images of participants, typical for each cluster.

**Figure 4:**
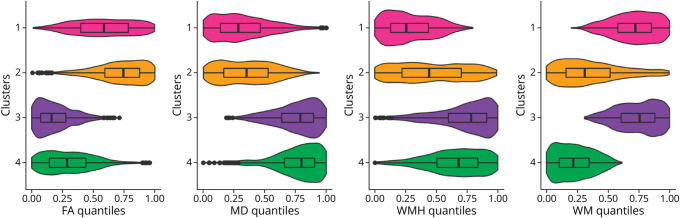
Distributions of Imaging Markers Across Clusters Violin plots showing the probability density function of white matter imaging markers for each cluster, with age-adjusted quantiles on the x-axis. Box plots are shown as overlay. FA = fractional anisotropy; MD = mean diffusivity; WM = white matter volume; WMH = white matter hyperintensities.

Figure 4 shows the distribution of the different WM imaging markers across clusters. For microstructural integrity and WM volume, clusters generally discriminated well between clusters, distinguishing at least 2 of 4 clusters from one another. Distributions were more overlapping for WMH volume, with a wide range of WMH especially in cluster 2.

The mean Adjusted Rand Index of 0.30 (SD = 0.07) suggested poor cluster stability. Nevertheless, visual inspection revealed that signatures, particularly for clusters 3 and 4, were quite stable across clustered subsamples, implying that etiologic insights can be validly derived from them.

The sensitivity analysis in which we performed clustering without age adjustment of imaging markers before clustering resulted in the identification of 4 clusters (eFigure 3). However, the cluster patterns were different than those with age adjustment before clustering. Without adjusting imaging markers for age, all clusters show a gradual increase of WM pathology with older age (eTable 1).

### Determinants of WM Clusters

**Figure 5:**
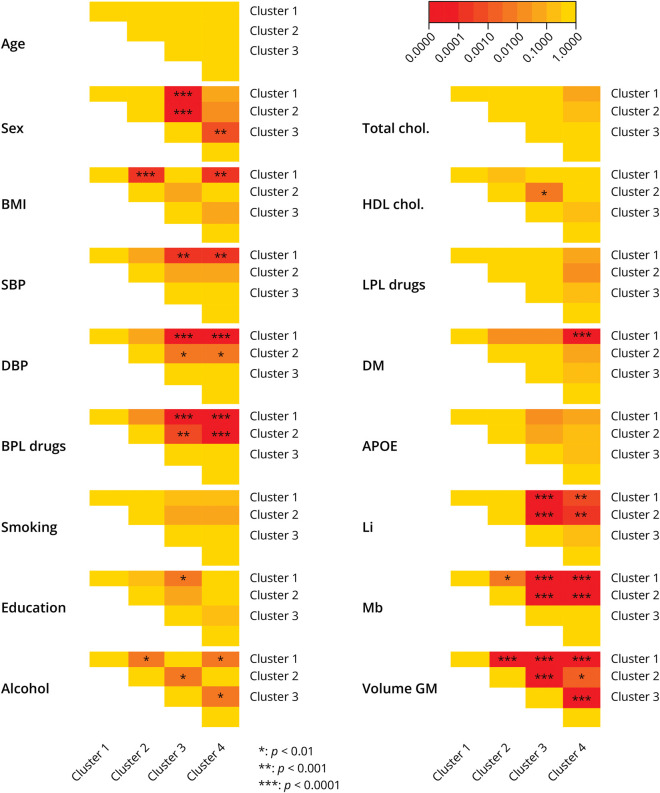
Pairwise Comparisons of Determinants Heatmap of *p*-values of pairwise comparisons among clusters for various determinants. BMI = body mass index; BPL = blood pressure lowering; chol = cholesterol; DBP = diastolic blood pressure; DM = diabetes mellitus; GM = gray matter; HDL = high-density lipoprotein; Li = lacunes; LPL = lipid lowering; Mb = microbleeds; SBP = systolic blood pressure.

Owing to the age standardization, clusters were well matched on age (Table 1 and Figure 5). Cluster 3 contained more women than the other clusters. Prevalence of cardiovascular determinants was generally lower in cluster 1 than in any of the other clusters, notably for hypertension, diabetes, and BMI. Concordantly, markers of small vessel disease were more prevalent in clusters 3 and 4 than in clusters 1 and 2. HDL cholesterol on average was slightly lower in cluster 2 than in cluster 3, whereas total cholesterol and use of lipid lowering medication did not differ between clusters. Smoking habits, alcohol consumption, educational attainment, and *APOE* genotype were also similarly distributed among clusters.

### Risk of Dementia, Stroke, and Mortality

Participants were followed up for a median 9.9 years for dementia (cluster 1: 9.9 years; cluster 2: 10.2 years; cluster 3: 9.6 years; cluster 4: 9.9 years), 10.8 years for stroke (cluster 1: 10.8 years; cluster 2: 10.9 years; cluster 3: 10.6 years; cluster 4: 10.6 years), and 10.8 years for mortality (cluster 1: 10.8 years; cluster 2: 10.9 years; cluster 3: 10.6 years; cluster 4: 10.7 years). During this follow-up, 291 participants developed dementia, 220 participants had a stroke, and 910 participants died. Associations between WM signatures and event risks of models 1, 2, and 3 are presented in eTable 2, eTable 3, and Table 2, respectively.

**Table 2:** Risk of Dementia, Stroke, and Mortality by White Matter Cluster

	Reference cluster			
	1	2	3	4
Dementia HR (95% CI)				
Cluster 1	—	**0.60 (0.42–0.85)**	**0.33 (0.23–0.47)**	**0.43 (0.30–0.63)**
Cluster 2	**1.67 (1.17–2.38)**	—	**0.55 (0.40–0.75)**	0.72 (0.52–1.00)
Cluster 3	**3.06 (2.12–4.42)**	**1.83 (1.34–2.51)**	—	1.33 (0.95–1.85)
Cluster 4	**2.31 (1.58–3.37)**	1.38 (1.00–1.91)	0.75 (0.54–1.05)	—
Stroke HR (95% CI)				
Cluster 1	—	0.74 (0.50–1.08)	**0.64 (0.42–0.98)**	**0.52 (0.35–0.77)**
Cluster 2	1.36 (0.93–1.98)	—	0.87 (0.60–1.28)	**0.70 (0.49–1.00)**
Cluster 3	**1.55 (1.02–2.37)**	1.14 (0.78–1.67)	—	0.80 (0.54–1.18)
Cluster 4	**1.94 (1.30–2.89)**	**1.43 (1.00–2.04)**	1.25 (0.85–1.84)	—
Mortality HR (95% CI)				
Cluster 1	—	**0.79 (0.65–0.94)**	**0.60 (0.49–0.74)**	**0.57 (0.47–0.69)**
Cluster 2	**1.27 (1.06–1.53)**	—	**0.77 (0.64–0.93)**	**0.72 (0.61–0.86)**
Cluster 3	**1.65 (1.35–2.03)**	**1.30 (0.08–1.57)**	—	0.94 (0.77–1.14)
Cluster 4	**1.76 (1.44–2.15)**	**1.38 (1.16–1.65)**	1.06 (0.87–1.30)	—

Abbreviations: HR = hazard ratio.

HRs of model 3, which are adjusted for age, sex, education, *APOE* genotype, lacunes, microbleeds, and cardiometabolic risk factors. Each cluster is used as reference once for determining HRs. Bold font indicates significant estimates.

Risk estimates were generally higher for dementia than for stroke and mortality. In fully adjusted models, dementia risk was increased most for cluster 3 (HR 3.06, 95% CI 2.12–4.42), followed by cluster 4 (HR 2.31, 95% CI 1.58–3.37), and to a lesser extent cluster 2 (HR 1.67, 95% CI 1.17–2.38), compared with cluster 1. In addition, clusters 1, 2, and 3 showed to be statistically distinct from one another in dementia risk, while cluster 4 was only statistically different from cluster 1.

Compared with cluster 1, incidence of stroke was higher for clusters 3 (HR 1.93, 95% CI 1.27–2.92) and 4 (HR 2.26, 95% CI 1.52–3.36), which remained significant after further adjustment for cardiovascular risk factors (Table 2). Mortality risk was increased for clusters 2, 3, and 4 compared with cluster 1, with significant differences between all clusters except between clusters 3 and 4 (Table 2).

Fully adjusted Cox proportional hazard models using the clustered data had lower AIC values than similar models using individual WM imaging markers for dementia risk (AIC >4,122.16), indicating a superior goodness of fit for the cluster model (AIC = 4,117.01). By contrast, models incorporating only WMH (AIC = 3,423.31) as independent variables for incident stroke showed a better goodness of fit compared with the cluster model (AIC = 3,426.58). For mortality risk too, the cluster-based model (AIC = 13,541.00) yielded a lower AIC value than the models using individual WM imaging markers (AIC >13,547.95) (eTable 4).

Further adjustment for gray matter volume showed broadly similar patterns, although it led to slightly higher effect estimates for cluster 2 on dementia and cluster 4 on mortality (eTable 5).

## Discussion

In this study, we demonstrate that heterogeneity in imaging markers of WM in the general population can be clustered in etiologically meaningful WM signatures based on MRI data. Differences between clusters in determinants and clinical outcomes suggest different underlying pathologies, which may have utility for risk stratification and targeted intervention.

Clustering methods are commonly used in brain MRI research for tissue segmentation on a voxel level, but as we have shown, they can be applied also to identify distinct WM signatures based on structural and diffusion MRI. Unraveling WM signatures, above and beyond investigating individual imaging markers, can be helpful to identify interrelated pathophysiologic processes and interactions between individually measured markers. Previous studies that used clustering on MRI markers generally focused on cognitive decline or other clinical outcomes in specific patient populations. Consequently, these studies considered a broader range of biomarkers for clustering, including CSF in addition to gray matter and WM traits on brain imaging.^9-11^ Although this may be useful for outcome prediction, we focused on imaging markers of cerebral WM to gain insight specifically in the etiologic role of WM pathology. The validation against several clinical outcomes underlines that differences between such signatures can be clinically meaningful, in particular regarding dementia. This may reflect a more complex role of WM pathology in development of dementia, as compared with stroke or mortality.

The variation between clusters in underlying pathology is further supported by differences in several clinical determinants. Vascular pathology was a hallmark of clusters 3 and 4, coinciding with pronounced reduction of microstructural integrity and the largest WMH volume. These findings align with the notion that reduced WM integrity and WMH burden are at least in part attributable to cerebrovascular disease.^22-25^ Of interest, *APOE* genotype was similar across clusters, suggesting a limited effect on WM signatures as identified in this study. However, previous research linked APOE to WMH volume in subcortical regions, but not periventricular,^26^ which could explain the absence of *APOE* differences because we did not include spatial information of WMH.

The identified signatures show that deterioration of a single WM imaging marker does not necessarily imply the deterioration of other WM imaging markers. This stands in contrast to clusters formed through clustering without prior adjusting imaging markers for age, where cluster patterns reveal a gradual increase in WM pathology with older age. This confirms our hypothesis that adjusting imaging markers for age was necessary to unravel heterogeneity of imaging markers and to avoid clusters primarily driven by age. This heterogeneity could be related to temporal evolution of pathology, which remains to be determined in future studies using longitudinal imaging data. Based on previously reported changes in brain MRI markers over time,^27-29^ it is plausible that individuals from cluster 1 may later shift to cluster 2, 3, or 4, and individuals from clusters 2 and 3 may shift to cluster 4. Although it is interesting to investigate the stability of cluster type over time, this aspect was beyond the scope of our study and remains to be explored in future research. Some of the observed differences, however, do not seem explicable solely by temporal changes. An interesting observation in that respect is the higher risk of dementia in clusters 3 and 4, despite difference in WM atrophy (substantial in cluster 4, but not in cluster 3). The dementia risk difference was only partially explained by smaller gray matter volumes in cluster 3, indicating an increased risk of dementia.^30,31^ It raises the question whether these clusters may reflect different etiologic subtypes of dementia and could be further investigated through cluster correlation with disease-specific biomarkers. Alternatively, we cannot rule out that WM volume segmentations might be influenced by coinciding gray matter atrophy.

Strengths of our study include the long-term, prospective ascertainment of dementia, stroke, and mortality in a large, population representative sample with multimodal brain MRI. Despite the new insights from the clustering approach, it is important to acknowledge the methodological imperfections and future challenges. First, some of the imaging marker values within clusters, particularly WMH burden in cluster 2, exhibited broad distributions. This might explain the relatively poor Adjusted Rand Index across subsamples, as WM imaging patterns in clusters 3 and 4 were more stable than clusters 1 and 2. However, the goal of our clustering was not to assign individuals perfectly into clusters, but to infer etiologic clues from WM signatures; in this respect, the various iterations of clustering provided rather consistent results based on visual inspection of WM signatures. Second, we only included global markers of brain structure. Future studies may also benefit from spatial information of imaging markers. Previous studies have already shown that spatial information of WMH is associated with different underlying pathologies and specific cognitive deficits.^32,33^ This could also apply for WM atrophy and microstructural deterioration (e.g., tract-specific FA and MD). Moreover, for similar volume of WMH, different locations may lead to different global FA or global MD values due to some regions of WM having a larger effect on the global FA or MD than other regions. Third, scans with cortical infarcts, causing tissue segmentation issues, had to be excluded, potentially introducing selection bias. Based on the observed clusterification, these individuals are expected to be in cluster 3 or 4, possibly leading to slightly underestimated disease risks of these clusters in the current analyses. Fourth, although >80% of individuals who attended the study center also underwent brain MRI, the selection may have led to a somewhat more healthy study population. Fifth, we have not externally validated the presented WM signatures.

In conclusion, heterogeneity in imaging markers of WM in the general population can be clustered in distinct and etiologically meaningful WM signatures based on MRI data. Differences among clusters in determinants and clinical outcomes suggest different underlying pathologies, which adds to understanding of disease pathophysiology, and may hold future utility for risk stratification.

## Appendix Authors

**Table:**

Name	Location	Contribution
Mathijs T. Rosbergen, MSc	Department of Epidemiology, and Department of Radiology and Nuclear Medicine, Erasmus MC University Medical Center, Rotterdam, the Netherlands	Drafting/revision of the manuscript for content, including medical writing for content; study concept or design; analysis or interpretation of data
Frank J. Wolters, MD, PhD	Department of Epidemiology, and Department of Radiology and Nuclear Medicine, Erasmus MC University Medical Center, Rotterdam, the Netherlands	Drafting/revision of the manuscript for content, including medical writing for content; study concept or design; analysis or interpretation of data
Elisabeth J. Vinke, PhD	Department of Epidemiology, and Department of Radiology and Nuclear Medicine, Erasmus MC University Medical Center, Rotterdam, the Netherlands	Drafting/revision of the manuscript for content, including medical writing for content; study concept or design; analysis or interpretation of data
Francesco U.S. Mattace-Raso, MD, PhD	Department of Epidemiology, and Department of Internal Medicine, Erasmus MC University Medical Center, Rotterdam, the Netherlands	Drafting/revision of the manuscript for content, including medical writing for content; study concept or design; analysis or interpretation of data
Gennady V. Roshchupkin, PhD	Department of Epidemiology, Department of Radiology and Nuclear Medicine, and Department of Medical Informatics, Erasmus MC University Medical Center, Rotterdam, the Netherlands	Drafting/revision of the manuscript for content, including medical writing for content; study concept or design; analysis or interpretation of data
Mohammad Arfan Ikram, MD, PhD	Department of Epidemiology, Erasmus MC University Medical Center, Rotterdam, the Netherlands	Drafting/revision of the manuscript for content, including medical writing for content; study concept or design; analysis or interpretation of data
Meike W. Vernooij, MD, PhD	Department of Epidemiology, and Department of Radiology and Nuclear Medicine, Erasmus MC University Medical Center, Rotterdam, the Netherlands	Drafting/revision of the manuscript for content, including medical writing for content; study concept or design; analysis or interpretation of data

## Glossary

AIC: Akaike Information Criterion
BMI: body mass index
DTI: diffusion tensor imaging
FA: fractional anisotropy
FLARIR: fluid-attenuated inversion recovery
GMS: Geriatric Mental State Schedule
HDL: high-density lipoprotein
HR: hazard ratio
MD: mean diffusivity
MMSE: Mini-Mental State Examination
WM: white matter
WMH: WM hyperintensity
